# Direct intensive care costs of severe sepsis and septic shock patients in Thailand

**DOI:** 10.1186/cc14097

**Published:** 2015-03-16

**Authors:** B Khwannimit, R Bhurayanontachai

**Affiliations:** 1 Songklanagarind Hospital, Hat Yai, Thailand

## Introduction

Costs of severe sepsis care from middle-income countries are lacking. This study investigated direct ICU costs and factors that could affect the financial outcomes.

## Methods

A prospective cohort study was conducted in the medical ICU of a tertiary referral university teaching hospital in Thailand over a 4-year period.

## Results

**Figure 1:**
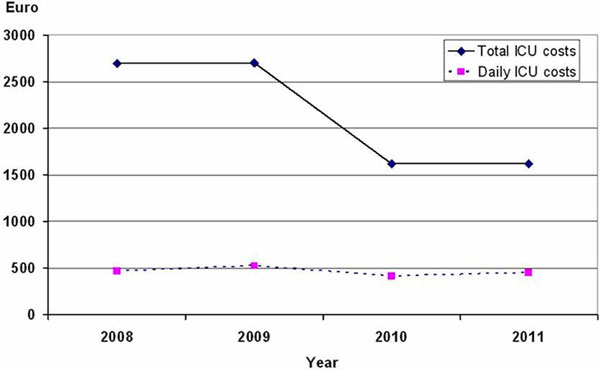
**Median ICU costs per patients and ICU cost per day according to study years**.

A total of 897 patients, with 683 (76.1%) having septic shock. Overall ICU mortality was 38.3%. The median (interquartile range) ICU length of stay (LOS) was 4 (2 to 9) days. Community, nosocomial and ICU-acquired infection were documented in 574, 282 and 41 patients, respectively. The median ICU costs were €2,067.2 (986.3 to 4.084.6) per patient and €456.6 (315.3 to 721.8) per day. The ICU costs accounted for 64.7% of the hospital costs. In 2008 to 2011, the ICU costs significantly decreased by 40% from €2,695.7 to €1,617, whereas the daily ICU costs decreased only 3.3% from €463.9 to €448.7 (Figure 1). The average ICU costs of patient with nosocomial and ICU-acquired infection were significantly higher than patients with community-acquired infection. By multivariate logistic regression analysis, age, nosocomial or ICU infection, admission from emergency department, number of organ failures, ICU LOS, and fluid balance in the first 72 hours were independently associated with total ICU costs.

## Conclusion

The ICU costs of severe sepsis management significantly declined in Thailand. However, the ICU costs were a financial burden accounting for two-thirds of the hospital costs. It is essential for intensivists to contribute a high standard of care within a restricted budget. The cost-effectiveness analysis should be evaluated in sepsis care cases.

